# Detection of polyandric behavior in giant Amazonian river turtle (*Podocnemis expansa*) using microsatellites markers

**DOI:** 10.1186/1753-6561-8-S4-P162

**Published:** 2014-10-01

**Authors:** Juliana Morais, Izeni Farias, Robinson Botero-Arias, Cristiane Araújo, Cássia Camillo, Cleiton Fantin

**Affiliations:** 1 Universidade do Estado do Amazonas, São José, AM, Brazil; 2 Universidade Federal do Amazonas, Manaus, AM, Brazil; 3 Instituto de Desenvolvimento Sustentável Mamirauá, Fonte Boa, AM, Brazil

## Background

In some species, presence of multiple paternity, due to polyandric behavior have important consequences in the effective size of a population when compared to unique paternity, mainly when it is about endangered species [1]. Because the exacerbated exploitation of meat, guts and eggs as food by local communities, giant Amazonian river turtle (*Podocnemis expansa*) is at low risk/dependant of conservation, according IUCN. Facing this, studies related to reproductive behavior of this specie have a great importance for contributing with programs about its conservation. Studies related to polyandric behavior, already evidenced in *P. expansa*, describe genetic benefits for the specie, because together with multiple paternity occurrence, it rises the genetic variability from offsprings and decreases the occurrence of endogamy between individuals [2,3]. The present study aims to verify the existence of polyandric behavior in females of Amazon turtles from Mamirauá Reserve of Sustainable Development (RDSM), using microsatellite markers.

## Materials and method

120 just-hatched individuals from four nests were analyzed, previously collected from RDSM. Samples of until 500µL of blood were collected by femoral vein puncture by using 1mL syringes and stored in microtubes with 500µL of absolute ethanol at 4ºC. After blood sampling, offsprings were released in the site of origin. DNA extraction was performed by CTAB method suitable for nucleated blood cells. After extraction, DNA was submitted to Polymerase Chain Reaction (PCR) following the economic protocol described by Schuelke [4]. Four microsatellites loci were used (Puni1D12, Pe344, Pe519 and Puni1E1) developed for the specie. PCR products were subjected to genotyping according DeWoody protocol [5], performed by an automatic DNA sequencer ABI 3130xl. Observed alleles analysis for each locus was performed by using GeneMaker v2.2.0 program, in order to identify the genotype of each locus from individuals sampled. Analysis for multiple paternity was done using the minimum method of Alleles Counting.

## Results and conclusion

When separately analyzed, four loci in all nests indicated multiple paternity, with at least three fathers contributing in each nest. In three nests the most polymorphic locus showed fourteen alleles and one nest had eighteen alleles. The least polymorphic locus, otherwise, showed eight alleles in two nests, seven in one nest and five in the last nest. Such results corroborates with previous studies which support as prevalent the polyandric behavior for *P. expansa*. RDSM population was considered ecologically extinct, by the high levels of predation in the past centuries and the low number of females spawning nowadays. From late 90s, nesting areas began to be protected in different areas from RDSM by local population. Presence of multiple paternity in similar conditions to most abundant populations can be considered a recovery signal of RDSM population. Results showed are part of the "Conservação de Vertebrados Aquáticos Amazônicos" project (Conservation of Amazon Aquatic Vertebrates), developed by Mamiraua Institute of Sustainable Development and sponsored by Petrobras, through "Programa Petrobras Ambiental" (Ambiental Petrobras Program).

## Sponsors

Mamiraua Institute of Sustainable Development, Ministério da Ciência, Tecnologia e Inovação-MCTI, Cnpq and FAPEAM.
